# Biological data derived from European weather radars

**DOI:** 10.1038/s41597-025-04641-5

**Published:** 2025-02-28

**Authors:** Peter Desmet, Judy Shamoun-Baranes, Bart Kranstauber, Adriaan M. Dokter, Nadja Weisshaupt, Baptiste Schmid, Silke Bauer, Günther Haase, Bart Hoekstra, Pieter Huybrechts, Hidde Leijnse, Nicolas Noé, Stijn Van Hoey, Berend Wijers, Cecilia Nilsson

**Affiliations:** 1 Research Institute for Nature and Forest (INBO), Brussels, Belgium; 2 Institute for Biodiversity and Ecosystem Dynamics, University of Amsterdam, Amsterdam, The Netherlands; 3 Cornell Lab of Ornithology, Cornell University, Ithaca, USA; 4 Finnish Meteorological Institute (FMI), Helsinki, Finland; 5 Swiss Ornithological Institute, Sempach, Switzerland; 6 Swiss Federal Institute for Forest, Snow and Landscape Research WSL, Birmensdorf, Switzerland; 7 Department Environmental Systems Science, Federal Institute of Technology (ETH), Zurich, Switzerland; 8 Swedish Meteorological and Hydrological Institute (SMHI), Norrköping, Sweden; 9 Royal Netherlands Meteorological Institute (KNMI), De Bilt, The Netherlands; 10 The Binary Forest, Braine-l’Alleud, Belgium; 11 Fluves, Ghent, Belgium; 12 Department of Biology, Lund University, Lund, Sweden

**Keywords:** Ecology, Climate sciences

## Abstract

Weather radars detect more than weather, they also continuously register the movements of billions of animals aloft in the lower atmosphere. This makes archived, unfiltered weather radar data a goldmine for biological monitoring purposes, providing coverage of the aerial habitat in a way no other method can. Here we present two datasets of biological data extracted from European weather radar data, obtained through a collaboration with the Operational Programme for the Exchange of Weather Radar Information (OPERA) and three national meteorological services. The datasets were created by processing weather radar data with methods optimized for extracting bird targets, resulting in vertical profiles of biological targets. The datasets collectively cover 141 radar stations in 18 countries, from 2008 to 2023. Data quality and coverage differs between years, countries, and radar stations, so care must be taken when evaluating data for each specific use case. Despite these challenges the datasets are currently the most comprehensive of their kind in Europe and open new avenues in understanding continental scale movements of aerial animals.

## Background & Summary

Since the second world war, the airspace across the world has been monitored by an ever-growing network of silent sentinels, constantly interrogating the skies. Specialized radars monitor all forms of movement in the air, and one of the most widespread networks globally is that of weather surveillance radars. Their main purpose is to quantify meteorological phenomena such as precipitation and monitor storm systems, but weather radars also measure other things aloft in the lower atmosphere. As our abilities to analyze and store the huge amounts of data gathered by weather radar networks increase, new possible applications arise.

One application that has seen a steep rise in use is the extraction of biological targets from weather radar data, information that is immensely useful for a broad variety of fundamental and applied biological and ecological questions^1,2^. As weather radars detect all aerial fauna, from insects^3^ to birds^4^ and bats^5^, they can continuously monitor the movements of animals in the air. Such continuous biodiversity monitoring is increasingly recognized as essential for detecting biodiversity loss and testing the efficacy of management measures and policies^6,7^, yet difficult and costly to obtain with traditional observation methods^1^.

Probably the greatest asset of using weather radar data in biological studies is the large temporal and spatial scopes that can be investigated^6^. For the first time we can directly measure several large scale phenomena, such as the continent-wide migration of birds^8,9^. Billions of birds traverse the earth each year, with far-reaching consequences for conservation, ecosystem health, disease spread, aviation safety and infrastructure development^6^. One limitation of using weather radar data for biological monitoring is the low taxonomic resolution, as targets often cannot be identified beyond their class (for example “bird” or “insect”). However, citizen science and other field observations can complement radar data with species-specific information^10^. There are also some noteworthy examples where further identification is possible due to specific animal behaviors, such as emergence of Brazilian free-tailed bats (*Tadarida brasiliensis*) at Bracken cave^5^, roosting swallows in the US^11^, and twilight flights of Common swifts (*Apus apus*)^12^. The overall low taxonomic resolution means that applications have been largely macro-ecological^13^ in nature, focused on aspects such as reducing threats along migratory flyways^14^ or monitoring changes to ensembles of species^9,15,16^.

The benefits of monitoring large parts of migration flyways and different habitats crucially relies on combining data from a network of weather radars, ideally over long periods. This is comparatively straightforward in overall homogeneous radar networks and where data are managed by a single agency. In the US, for example, NOAA (National Oceanic and Atmospheric Administration) provides public access to archived Level II data (radar sweeps for base and dual-polarization quantities) that can be used to derive meteorological and biological products^17^. Open access to this archive has greatly facilitated the use of weather radar data in biology and led to the emergence of a vibrant field of aeroecology, the study of life in the air^18,19^. In contrast, the European network of weather radars is heterogeneous in hardware, data processing and data availability, with national meteorological services operating with varying policy priorities and under very different conditions, which has long hampered the development of a joint dataset of biological weather radar data from across Europe^20^. However, with careful consideration and quality control, pan-European comparisons can be made^8,21,22^.

Here we present two public datasets of animal movement data derived from European weather radars. They are the result of long-term interdisciplinary collaborations through the COST action ENRAM (European Network for the Radar surveillance of Animal Movement)^23^ and BiodivERsA projects GloBAM and HiRAD. The datasets were created by processing radar data with dedicated and validated methods for detecting birds and especially bird migration, resulting in summaries of movement characteristics (e.g. density, direction and speed) at regular altitude intervals across the lower atmosphere. Despite heterogeneity in coverage and data quality, they provide the largest and most complete datasets of their kind in Europe. By creating, standardizing and publicly archiving these data, we reduce computing and access difficulties to facilitate aeroecology research and applications. The datasets are deposited on Zenodo and can also be explored in the Aloft data portal (see Aloft data portal).

## Methods

The data were created by processing polar volume data (**PVOL**) to vertical profiles of biological targets (**VP**). These are then combined into vertical profile time series (**VPTS**).

### PVOL data access

PVOL data result from the scanning routine of a radar (also known as volume coverage pattern or VCP). It consists of a number of scans (also called sweeps) collected at different elevation angles (angle between radar scan and horizon), with quantities observed by the radar such as reflectivity factor (DBZ) and radial velocity (VRAD) (see for example^24^ for details). Scanning routines are typically conducted every 5 to 15 minutes and generally result in one PVOL file per routine.

To exchange such data, national meteorological services in Europe collaborate in OPERA, the Operational Programme for the Exchange of Weather Radar Information^25^, under the umbrella of the European Meteorological Services Network, EIG EUMETNET. OPERA member countries submit PVOL data (or scans) to OPERA in real-time, using the ODIM HDF5 format^26^. Data are sometimes cleaned (i.e. filtered) for meteorological purposes prior to distribution (see Usage Notes and^20^). These data are subsequently used in OPERA production centers to produce meteorological products. They are also forwarded to the OPERA development environment (ODE) server, where software called the BALTRAD Toolbox^27^ does additional processing and archiving. This server is maintained by the Swedish Meteorological and Hydrological Institute (SMHI) and further referred to as “BALTRAD”.

European PVOL data are generally not publicly available. Access is granted to members of the ENRAM consortium (including authors of this paper) by meteorological institutes of many OPERA member countries (i.e. the 18 countries covered by the data, as well as Austria and the United Kingdom) through a license agreement. It states that PVOL data cannot be distributed further and can only be used “for the purpose of extracting animal migration information for scientific research”. These restrictions do not apply to derived VP/VPTS data. For the *BALTRAD_VPTS*^28^ dataset, we obtained PVOL data from BALTRAD. For the *UVA_VPTS*^29^ dataset, the University of Amsterdam (UvA) obtained PVOL data as part of several research projects related to aviation safety and wind energy, from three national meteorological services: the Royal Meteorological Institute of Belgium (RMI), German Meteorological Service (DWD) and Royal Netherlands Meteorological Institute (KNMI). In contrast with (recent) data provided to BALTRAD, these data are unfiltered and often contain dual polarization quantities, which results in higher quality VP data (see Usage Notes).

### PVOL to VP data processing

We created VP data by processing PVOL data with the vol2bird algorithm^30,31^, using the default settings. A VP consists of estimated density, speed and direction of biological targets such as birds, bats and insects within a weather radar volume per altitude layer. The vol2bird algorithm uses a combination of local variation in radial velocity (*VRADH*) and reflectivity (*DBZH*) thresholds to extract animal (mainly bird) signals. Each PVOL file results in a corresponding VP file, a HDF5 file expressed in the ODIM bird profile format.

**Fig. 1:**
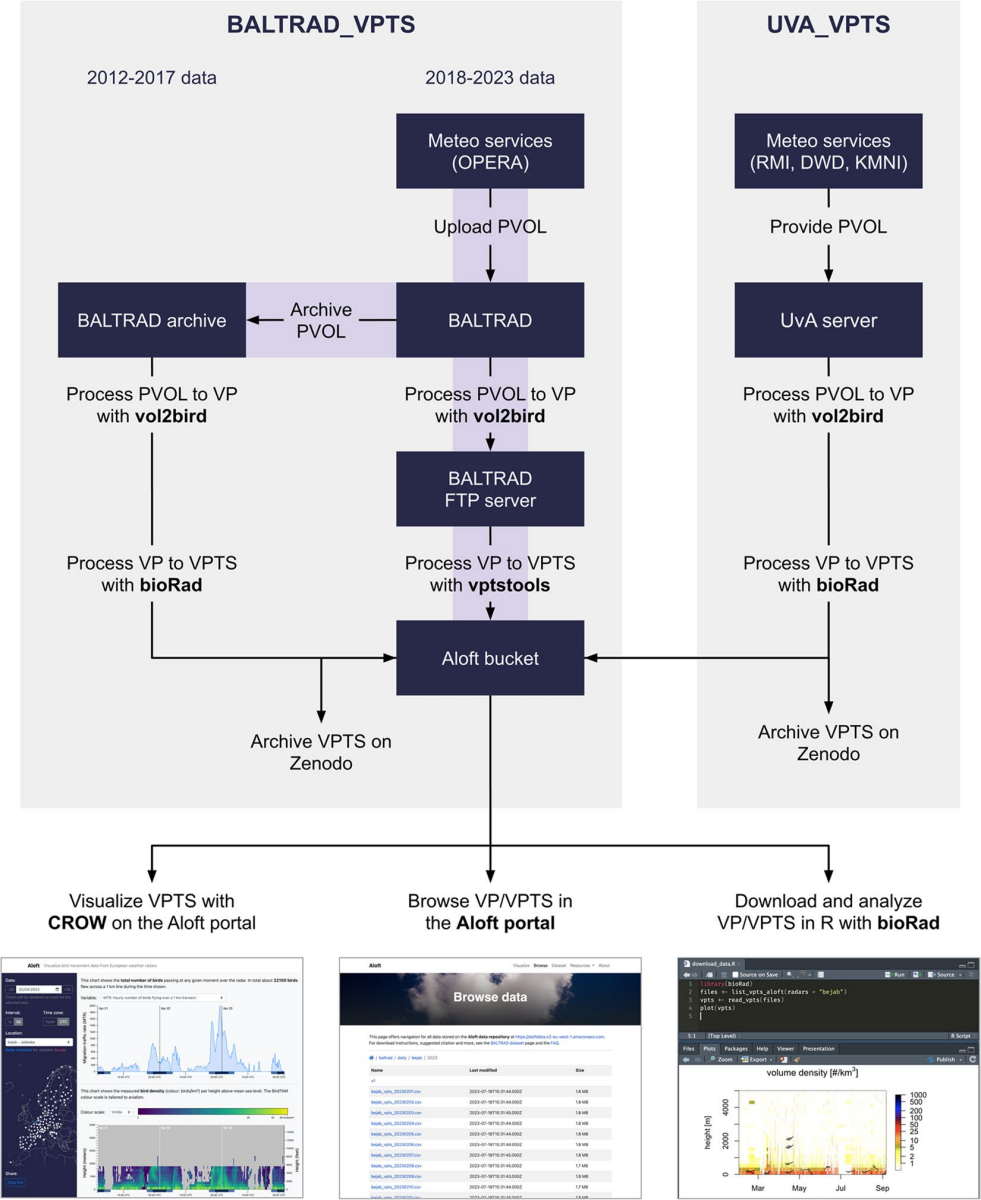
Schematic diagram of the processing steps used to create the datasets, showing the main data storage locations (boxes), processes (arrows) and access methods (screenshots). A number of operational services (purple) process incoming PVOL to VP and VP to VPTS on a daily basis.

For *BALTRAD_VPTS*, data from 2012 to 2017 were obtained from the BALTRAD archive and processed to VP data once, while data from 2018 onward are created by an operational service at BALTRAD (see Fig. 1). For *UVA_VPTS*, data were processed once. All processing was done with vol2bird v0.3 or v0.5. The vol2bird version and used parameters are recorded in the VP metadata.

### VP to VPTS data processing

We then extracted the tabular data and relevant metadata from the VP files (55 772 262 files), combined these into time series and converted those to the VPTS CSV (v1.0) format^32^. This format defines all measurements, facilitating standardized data exchange. While this process removes some metadata, it does not reduce the amount of data records, nor does it statistically aggregate data. *BALTRAD_VPTS* data from 2018 to 2023 were converted using the Python package vptstools^33^ as part of an operational service. All other data were converted using the R package bioRad^34,35^ (see Fig. 1).

We first created daily CSV files per radar (298 498 files) and then concatenated and gzipped these into monthly csv.gz files (10 991 files). The VPTS files are more storage efficient than the VP files, allowing faster downloads and analyses. For the same time period, daily files were on average 25% of the original VP file size, monthly files 6%.

### Long-term archiving

Finally, we performed a data format validation on all VPTS data using the Python library frictionless-py^36^ (see Technical Validation) and archived the monthly-packaged data on Zenodo, a free-to-use, general-purpose research repository. One deposit was created for each source (BALTRAD and UvA).

### Operational services

At the time of writing, data derived from BALTRAD are updated on a daily basis. Since 2015, SMHI has maintained a service that processes incoming PVOL to VP data and provides access to these via a restricted FTP server, where data older than two days are deleted. To facilitate access and prevent data loss, the Research Institute for Nature and Forest (INBO) maintains a service since 2017 that downloads the VP data from the FTP server to a publicly accessible Amazon S3 cloud object storage (further referred to as “bucket”). Since 2023, an additional service generates daily and monthly VPTS data from VP data (see Fig. 1). The bucket is gradually growing in size, providing public access within 48 hours after raw data were collected. Although the bucket and services are offered for free, their maintenance, computing and hosting is not. Those costs have been covered by a number of funding sources, but, unfortunately, do not guarantee long-term availability.

## Data Records

A static version of the data was deposited on 2025-01-21 on Zenodo as two datasets:

- BALTRAD_VPTS - Vertical profiles of biological targets derived from European weather radars: 10.5281/zenodo.14711024^28^
- UVA_VPTS - Vertical profiles of biological targets derived from weather radars in Belgium, Germany and the Netherlands: 10.5281/zenodo.14711244^29^

For other data formats and daily updates, see Aloft data portal.

### File properties, organization, naming and format

Both datasets contain vertical profile time series (VPTS). A VPTS expresses the density, speed and direction of biological signals (here predominantly birds) within a weather radar volume, grouped into altitude bins (here 200 m resolution/binsize) and measured over time (typically every 5 or 15 min). It can be expressed as tabular data containing three grouping properties (*radar*, *datetime*, *height*), calculated measurements (18 columns), radar metadata as found in the source VP file (4 columns) and the name of that source file (*source_file*). See Table 1 for a description of all properties.

**Table 1:** Overview of the properties (i.e. columns) in the datasets, including their name, description, data type and example value. Properties follow the VPTS CSV (v1.0) format. The example values are derived from radar SEANG in Ängelholm, Sweden, on 29 August 2020 at 20:30 UTC.

Name	Description	Data type	Example value
radar	Radar identifier.	string	seang
datetime	Nominal date and time of the measurement, as an ISO 8601 formatted string in UTC.	datetime	2020-08-29T20:30:00Z
height	Lower bound of the altitude bin in m above sea level. Also referred to as *HGHT* or *bin_lower*.	integer	800
u	Ground speed component west to east in m/s.	number	5.08662
v	Ground speed component south to north in m/s.	number	-7.49789
w	Vertical speed in m/s.	number	-9.347358
ff	Horizontal ground speed in m/s. Also referred to as *speed*.	number	9.060467
dd	Ground speed direction in degrees clockwise from north. Also referred to as *direction*.	number	145.8468
sd_vvp	VVP radial velocity standard deviation in m/s. Also referred to as *rmse*.	number	3.584702
gap	Angular data gap detected.	boolean	FALSE
eta	Animal reflectivity in cm^2^/km^3^. Also referred to as *linear_eta*.	number	79.07099
dens	Animal density in animals/km^3^.	number	7.188272
dbz	Animal reflectivity factor in dBZ.	number	-6.42906
dbz_all	Total reflectivity factor (bio + meteo scattering) in dBZ. Also referred to as *DBZH*.	number	11.28484
n	Number of data points used for the ground speed estimates (quantities *u*, *v*, *w*, *ff*, *dd*).	integer	732
n_dbz	Number of data points used for reflectivity-based estimates (quantities *dbz*, *eta*, *dens*).	integer	7771
n_all	Number of data points used for the radial velocity standard deviation estimate (quantity *sd_vvp*).	integer	1375
n_dbz_all	Number of data points used for the total reflectivity estimate (quantity *dbz_all*). Also referred to as *nbins*.	integer	9589
rcs	Radar cross section per bird in cm^2^.	number	11
sd_vvp_threshold	Lower threshold in radial velocity standard deviation (profile quantity *sd_vvp*) in m/s. Biological signals with *sd_vvp < sd_vvp_threshold* are set to zero. Defaults to 2 m/s for C-band radars and 1 m/s for S-band radars if not specified.	number	2
vcp	Volume coverage pattern, unitless. Documented on Wikipedia for NEXRAD. Also referred to as *scan_strategy*.	integer	
radar_latitude	Latitude of the radar location in decimal degrees, using the WGS84 datum. Constant for all records from the same *radar*.	number	56.3675
radar_longitude	Longitude of the radar location in decimal degrees, using the WGS84 datum. Constant for all records from the same *radar*.	number	12.8517
radar_height	Height of the center of the radar antenna in m above sea level. Constant for all records from the same *radar*.	integer	209
radar_wavelength	Wavelength of the radar in cm. Constant for all records from the same *radar*. Most C-band radars operate at approximately 5.3 cm wavelength, most S-band radars at 10.6 cm.	number	5.348661
source_file	URL or path to the source file from which the data were derived.	string	seang_vp_20200829T203000Z_0x81540b.h5

The files are organized as follows:

- country: a *<country>.tgz* file containing directories.
- radar: a *<radar>* directory.
- year: a *<yyyy>* directory.
- month: a *<radar>_vpts_<yyyy><mm>.csv.gz* file.

Where:

- *<country>*: 2-letter ISO 3166-1 alpha-2 code.
- *<radar>*: 5-letter ODIM NOD radar identifier, where the two first letters signify the country (ISO 3166-1 alpha-2 code) and the last three the radar station (e.g. SEANG for the Swedish radar at Ängelholm). See also Radar metadata.
- *<yyyy>*: year.
- *<mm>*: month (with leading zero).

Each monthly file uses the VPTS CSV v1.0 format (see Methods). The Table Schema for this format (*vpts-csv-table-schema.json*) is deposited with the data as technical documentation. All timestamps are in UTC.

### License

The data are released under a Creative Commons Zero waiver.

### Geographic coverage

**Fig. 2:**
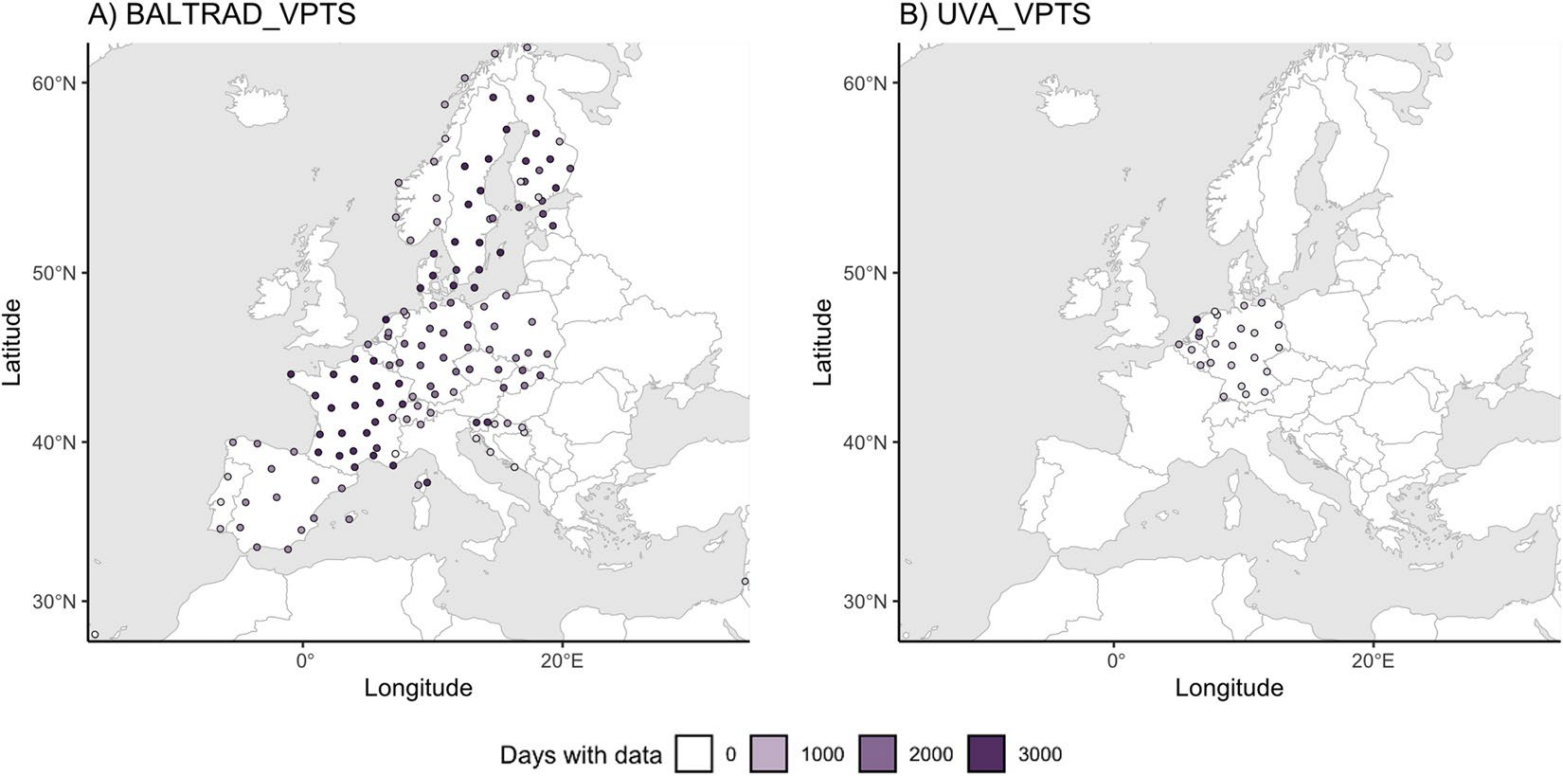
Maps of radar locations showing how many days with data are available (color) and the area used to extract biological data (size of circle, 25 km radius shown). Radars that changed code, but not location, are combined. (**A**) The 140 radar locations included in *BALTRAD_VPTS*. (**B**) The 24 radar locations included in *UVA_VPTS*.

*BALTRAD_VPTS*^28^ contains data from 151 radars at 140 locations in 18 countries, ranging from the Spanish radar ESLPA on Gran Canaria in the south (latitude 28.0) to NOHAS in arctic Norway in the north (latitude 70.6) and longitudinally, from -15.6 (ESLPA) to 34.8 (ILTLV) in Israel. *UVA_VPTS*^29^ contains data from 24 radars at 24 locations in three countries, with only BEZAV not included in *BALTRAD_VPTS*. Collectively, the datasets cover 152 radar stations at 141 locations (see Fig. 2).

Note that the datasets do not include all radar stations in Europe, but only those that are 1) included in the OPERA network, 2) where the managing meteorological service signed the license agreement with ENRAM and sends data to OPERA, and 3) where data contain the necessary properties to be processed to VP/VPTS data. Even if a radar station has data associated with it, not all locations can be reliably compared due to heterogeneity in data acquisition, cleaning and collation procedures before the biological data are extracted^20^. In recent years, many radars were upgraded from single polarization to dual polarization^37^. In some cases, this opportunity was used to move the radar to a more suitable location in close proximity to the original radar. For 11 radars in Germany and Sweden the radar code was also changed (see Table 2), resulting in a location being associated with two codes.

For each radar, data collected between 5 and 25 km from the radar was used to calculate VP data. This was updated to 5 and 35 km on 2019-09-23 for *BALTRAD_VPTS* and 2020-01-02 for *UVA_VPTS* to improve vertical profile coverage at higher altitudes while not compromising altitudinal resolution. The area used for biological monitoring is thus much smaller than the maximum range of the radar, which extends well beyond 200 km for most radars. Although biological signals can be detected further from the radar, the curvature of the earth and the elevation angle of the lowest beams cause the radar to “overshoot” low-flying fauna at distances further than 35 km (see e.g.^38^). The beam width also rapidly increases with range, leading to an effective loss in altitudinal resolution. The area closest to the radar (<5 km) is excluded as it does not give reliable data due to radar sidelobes and strong reflections from objects on the ground^39^ (ground clutter) obscuring bird movements.

### Temporal coverage

The datasets span 16 years, from 2008 to 2023 (2012 to 2023 for *BALTRAD_VPTS*^28^), covering 4 877 out of 5 844 possible days (83%). Depending on the scanning strategy, data are generally collected at 5 or 15 min intervals, resulting in 1 379 816 unique timestamps (floored to 5 min intervals). The number of days with data at each radar station varies greatly, spanning from almost full coverage at 4 684 days (NLDHL) to only 23 days (ESLPA). See the Supplementary Information for a visual overview of the number of days with data per source, radar and year.

Gaps in the temporal coverage of data occur due to a variety of reasons. Power outages or radars being offline for maintenance can lead to missing data at single locations^40^. BALTRAD or other processing service issues can lead to missing data across locations, also for extended periods of time. For most years in *UVA_VPTS* only data during migration seasons (February-May, August-November) are retained.

### Taxonomic coverage

Except for very specific circumstances (see Background & Summary), biological signals in weather radar data typically offer limited opportunities for species identification. The vol2bird algorithm uses different aspects of the return echos and the movement of objects in relation to the radar and to prevailing wind to extract biological signals^30^. However, some ambiguity will remain and background knowledge of the species composition and movements at different locations will be required to correctly interpret the data. Comparative analysis across different monitoring systems have been used to validate the method in for example^4,30,41,42^. The vol2bird algorithm is optimized for broad-scale nocturnal migration of small birds, and out of the box will work best in these situations^30^.

During the vol2bird processing a threshold is applied to the standard deviation of the residuals in the volume velocity profiling procedure, a method used to determine speed and direction of targets^34^. This threshold (*sd_vvp_threshold*) is used to separate self-propelled animals flying at heterogeneous airspeeds relative to the radar from rain and insects moving more uniformly with the wind. This threshold creates a classification trade-off: when set too low, rain may be included as animals; when set too high, animals may be excluded when movement directions and speeds are uniform. The default threshold (*sd_vvp_threshold*) in bioRad is set at 2 m/s for C band^30^ and 1 m/s for S band and can be adjusted after processing. By default, animal densities are converted from radar reflectivity using a radar cross section (*rcs*) of 11 cm^2^, which represents the average size of a nocturnal bird migrant in Europe, estimated in a field campaign in the Netherlands, Belgium and France that collocated a bird radar with several weather radars^30^. The *rcs* can be adjusted ad hoc, also after processing, using the bioRad function *rcs()*. Gaussian mixture models fit to VPTS can further refine the distinction between birds and insects^22^, especially in cases where increasing the *sd_vvp_threshold* would interfere with the bird signal extraction (see Gaps caused by uniform movements).

## Technical Validation

We validated the format of the datasets^28,29^, by comparing the data with the VPTS CSV v1.0 Table Schema (deposited as *vpts-csv-table-schema.json* with the data), which defines the expected columns, required values, data types and constraints. Except for 26 cases of *dbz_all* values above the expected 100 (in *BALTRAD_VPTS*’s *demem_vpts_202103.csv.gz*), all 1 353 818 545 rows of data were technically valid and should be readable without issue. Note that duplicate *radar*, *timestamp*, *height* combinations may occur: these are the result of duplicate source files or rounded timestamps in the PVOL data.

The European weather radar network is a heterogeneous network, with national meteorological services using radar hardware, signal processing software, scanning strategies, data cleaning and data storage protocols that vary across Europe. Data quality and interoperability therefore varies, mainly between countries but in some cases also between locations, and over time. Such differences can influence the VPTS data and care must be taken when comparing different countries, locations and time periods. We have performed several data quality assessments on the PVOL and VP data (mainly for *BALTRAD_VPTS*) and summarized the results by country in https://github.com/aloftdata/data-repository/wiki. Our manual quality and consistency checks do not cover the entire dataset. We therefore encourage users to evaluate qualitative and quantitative requirements for their use case and welcome them to report quality issues in the wiki. Some of the regularly encountered issues are listed below.

### Rain contamination

**Fig. 3:**
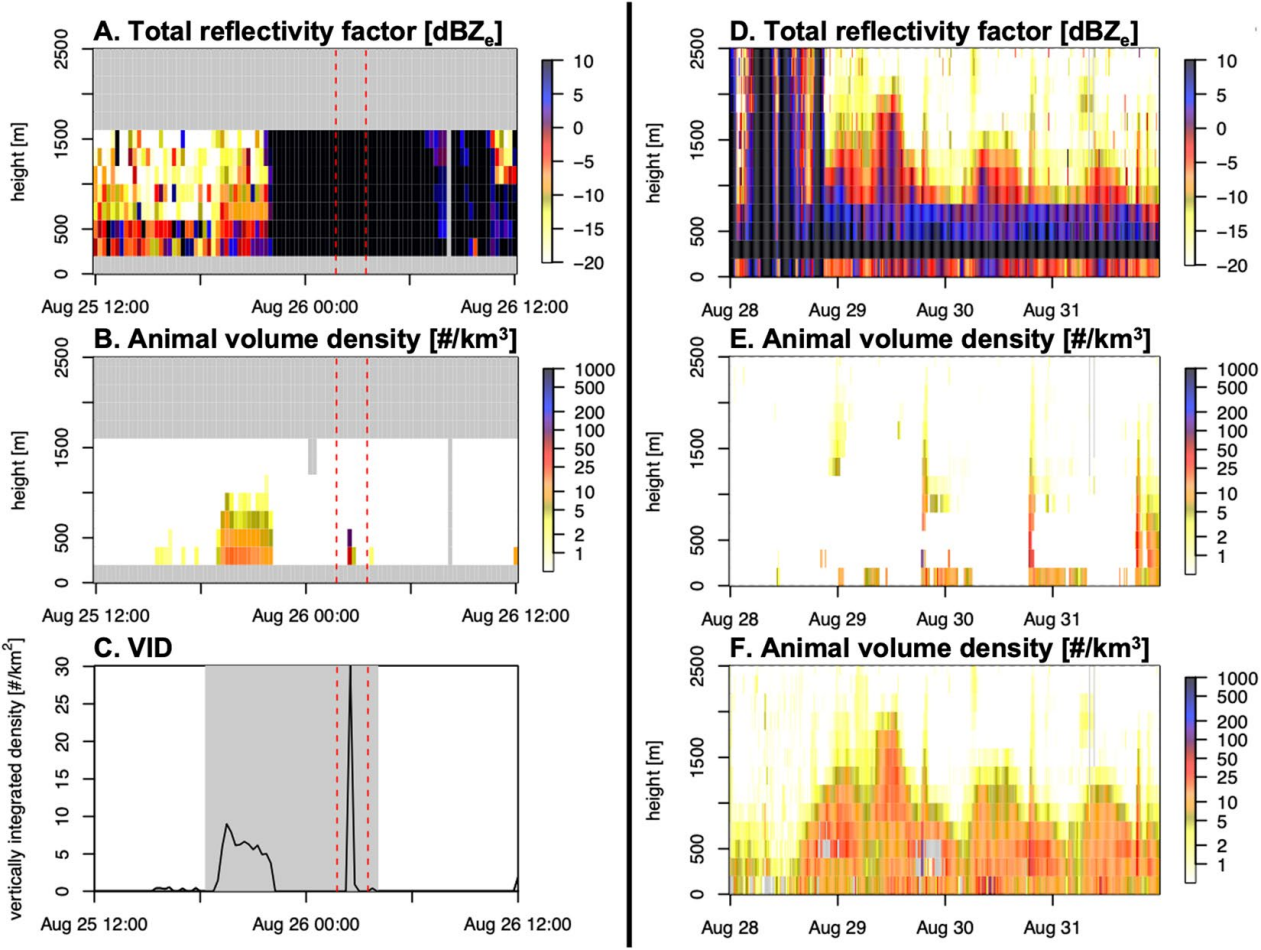
(**A**–**C**): Example of residual rain contamination in the biological density data, derived from radar SEANG in Ängelholm, Sweden, in August 2020. (**A**) Total reflectivity factor (*dbz_all*) over height. Profiles appear truncated above 1600 m. A rain event (black) is seen starting before midnight. (**B**) Reflectivity attributed to biological targets over the same period. Nocturnal migration is seen before the rain event, with a typical gradual increase and decrease. Later in the night there is a clear sign of rain contamination (between dotted red lines), with two altitude bins temporarily showing very high densities. (**C**) Vertical integration of the above biological density. The large peak in vertically integrated density is due to the high density of the rain contamination event. (**D**–**F**): Example of gaps due to a high radial velocity standard deviation threshold (*sd_vvp_threshold*), derived from radar SEHEM in Hemse, Sweden, in August 2021. (**D**) Total reflectivity factor (*dbz_all*) over a period of intense migration. (**E**) Animal volume density using the default *sd_vvp_threshold* of 2. This high threshold filters out animals moving uniformly in the same direction, resulting in gaps (white). (**F**) Animal volume density using a lower *sd_vvp_threshold* of 0.5, retaining more of the migration dynamics. Lowering the threshold retains more nocturnal biological scattering (likely a mixture of birds and insects), but also retains substantial diurnal reflectivity, which are likely due to insects, especially in this early migration season example when insects are still relatively active.

Even though the vol2bird algorithm can separate biological and meteorological targets with high accuracy^30^, some rain contamination may persist in the biological data. Rain has a very high reflectivity, resulting in a disproportionately large effect on the derived biological density when miss-classified as biological targets. This will often occur either directly before or after precipitation events, and may be caused by miss-classifying a single rain-filled range gate (a small volume of airspace measured by the radar) as birds (see Fig. 3). Rain contamination is usually easy to visually identify in VPTS plots, as high total reflectivity (*dbz_all*, also referred to as *DBZH*) extending from the ground to several km above ground or as isolated altitude bins with very high biological densities (*dens*). Rain contamination can also be spotted by plotting and comparing animal density (*dens*) and total reflectivity (*dbz_all*) (see Fig. 3) and events may be excluded based on characteristic altitudinal patterns of *dbz_all* (see e.g.^8,15^).

### Gaps caused by uniform movements

Especially during very intense and highly uniform movements of migratory birds, and during bird migration events with strong insect contamination, detected bird speeds and directions will become more spatially homogeneous and radial velocity standard deviation (*sd_vvp*, see^30,34^) might approach that of wind-borne insect movements. A default *sd_vvp_threshold* (2 m/s) will filter those out, resulting in characteristic “holes” when plotting the VPTS data (see Fig. 3E,F, for details see Taxonomic coverage). The *sd_vvp_threshold* can be adjusted in bioRad with the function *sd_vvp_threshold()*. Nussbaumer *et al*.^22^ specifically addressed the gaps by omitting the *sd_vvp_threshold* on rain-free VPs and instead applied Gaussian mixture models to correct for the presence of insects.

### Truncated vertical profiles

VPs may appear truncated, with biological intensity artificially cut off at higher altitudes above the radar, resulting in vertically integrated densities being underestimated (see Fig. 3). This issue was noted especially in several radars in Poland and France. Truncation at higher altitudes appears when the PVOL data only includes relatively low elevation scans, and thus the beam does not reach higher altitudes within the range used to extract VP data. If no additional elevation scans are available, extending the maximum range of PVOL processing can increase the covered altitudinal range. This approach was for example used in Nilsson *et al*.^8^.

### Filtering of biological signals prior to data exchange

In recent years, several countries have used dual-polarization to clean PVOL data of non-meteorological signals prior to data exchange with OPERA, leading to largely empty VPTS data even when biological activity is present^20^. Cleaning policies differ between national meteorological services and may result in systematic differences in the magnitudes of biological signals. For example, most Spanish radar stations are located along a major migratory flyway but show almost no bird movement, and Estonian data shows relatively low bird densities compared to its neighboring country Finland.

### Noise from moving (man-made) objects

Reflections caused by truly static objects in the landscape, such as buildings and mountains, can be reliably removed using Doppler filtering by radar signal processors based on their velocity being close to 0 m/s. However, moving objects, such as waves or spinning wind turbines, are difficult to filter out using the same approach and might show a radar signature similar to animals, causing them to be classified as such. Depending on the nature of these objects and their movements, this might result in occasional spikes or higher background noise in the animal densities. With rapid expansion of wind energy production across Europe the amount of noise generated by wind parks is likely to increase over time, requiring careful consideration of its effects on temporal patterns derived from VPTS data for radar stations located close to wind parks.

## Usage Notes

### Aloft data portal

The easiest way to explore and visualize the data is through the Aloft data portal (https://aloftdata.eu). It provides access to the bucket that currently contains both datasets and daily updates of the BALTRAD data (see Operational services). The bucket organizes data in hierarchical directories: source (e.g. *baltrad*, *uva*), format (three formats: *hdf5* for VP, *daily*/*monthly* for VPTS), radar, year, month, and day. Files can be downloaded in the browser or by using a software tool that supports S3, such as AWS CLI or rclone, which allows users to maintain a synchronized local copy of the bucket’s (sub)directories. BALTRAD data can be visualized directly in the browser at https://crow.aloftdata.eu^43^. By selecting a date (or three-day interval) and radar, it visualizes both bird density at different heights and migration traffic rate (*MTR*).

### Recommended software

Data on Zenodo can be downloaded as one .tgz file per country, which contains the VPTS data as .csv.gz files (see Data Records). Once decompressed, data can be read with any CSV reader. For expected data types, see the associated *vpts-csv-table-schema.json* file, which can be read automatically with software supporting the Data Package standard.

For data analysis, we recommend using the R package bioRad^34^, which can directly read (compressed) VPTS data with *read_vpts()* and provides functionality to filter, time-regularize and plot data (*filter_vpts()*, *regularize_vpts()* and *plot()*, respectively). It can also vertically integrate data with *integrate_profile()* and calculate migration traffic rate (*MTR*) and vertically integrated density (*VID*) (see^34^ for an overview of these properties).

### Radar metadata

A list of radars can be found at https://aloftdata.eu/radars/, which is derived from a database maintained by OPERA and indicates radar code (ODIM identifier), location, coordinates, characteristics and status. This list can change over time as new radars are installed and old radars decommissioned (indicated with status = 0). Codes are issued by OPERA member countries for new radars and sometimes replace those of existing radars after major hardware upgrades (see Geographic coverage, Table 2).

**Table 2:** Radar stations for which the radar code has changed.

Location	Previous code	Current code	Year(s) of change
Feldberg, Germany	DEFLG	DEFBG	2018/2019
Isen/München, Germany	DESNA	DEISN	2019
Åtvidaberg (Vilebo), Sweden	SEVIL	SEATV	2019
Hemse (Ase), Sweden	SEASE	SEHEM	2017
Hudiksvall, Sweden	SEHUD	SEHUV	2016/2017
Karlskrona, Sweden	SEKKR	SEKAA	2018/2019
Kiruna, Sweden	SEKIR	SEKRN	2020/2021
Luleå (Rosvik), Sweden	SELUL	SELLA	2018/2019
örnsköldsvik, Sweden	SEOVI	SEOER	2017
östersund, Sweden	SEOSU	SEOSD	2016
Vara, Sweden	SEVAR	SEVAX	2015/2016

### Use cases

The data presented here have the potential to be used in a wide range of applied and fundamental ecological studies. When the heterogeneity in data is appropriately addressed (see Technical Validation) and the limited taxonomic resolution recognized (see Taxonomic coverage) these data can be used to map the mass movements of animals in the air across Europe^8,21,22^. Applications include investigating anthropogenic disturbances^44,45^, disease spread^46^, aviation safety^47,48^, fundamental characteristics of animal migration^15,49^ and reactions to extreme events^50,51^. For more details on possible applications, see for example^2,6^.

### Future developments

OPERA is currently changing how radar data are archived at the European level, which will likely lead to changes in the data flow to BALTRAD and the bucket. Should these changes by OPERA also include that only heavily cleaned data are archived centrally, it would become impossible to extract biological signals^20^ across the European network without working with each national meteorological service individually, and a unique tool for large scale biodiversity monitoring will be lost. However, with a willingness to accommodate data use beyond meteorology, the current adjustments to the OPERA processing pipeline could lead to more homogeneous products with better access to uncleaned data, which would greatly benefit biological monitoring, applications such as aviation safety or wind farm operations and society as a whole. The value of long-term monitoring datasets in biology cannot be stressed enough. It is our hope that the increased spread and use of the data described here will encourage more policy makers to appreciate the value of reusing already collected radar data, which is only made possible by archiving and sharing uncleaned radar products. See Shamoun-Baranes *et al*.^1,20^ for more details.

**Table 3:** Software tools and data formats that were used to create the datasets. The overview includes the software language, used version(s) and link to the open source repository.

Name	Language	Version	Repository URL
vol2bird	C	0.3 and 0.5^31^	https://github.com/adokter/vol2bird
bioRad	R	0.7^35^	https://github.com/adokter/bioRad
vptstools	Python	0.3^33^	https://github.com/aloftdata/vptstools
frictionless-py	Python	5.15^36^	https://github.com/frictionlessdata/frictionless-py
CROW	Javascript	1.3^43^	https://github.com/aloftdata/crow/
ODIM bird profile	(data format specification)	August 17, 2021	https://github.com/adokter/vol2bird/wiki/ODIM-bird-profile-format-specification
VPTS CSV	(data format specification)	1.0^32^	https://github.com/aloftdata/vpts-csv

## Supplementary information

Supplementary Information

## Data Availability

We used a number of software tools and data formats to create, process, validate and visualize the datasets, listed in Table 3 (see also Fig. 1 and Methods). All of these are open source and with the exception of frictionless-py maintained by the authors.
