# Supplementary figures and images for: Biological data derived from European weather radars
Source: Sci Data. 2025 Feb 28;12:361. doi: 10.1038/s41597-025-04641-5 (PMC11871220; doi:10.1038/s41597-025-04641-5)

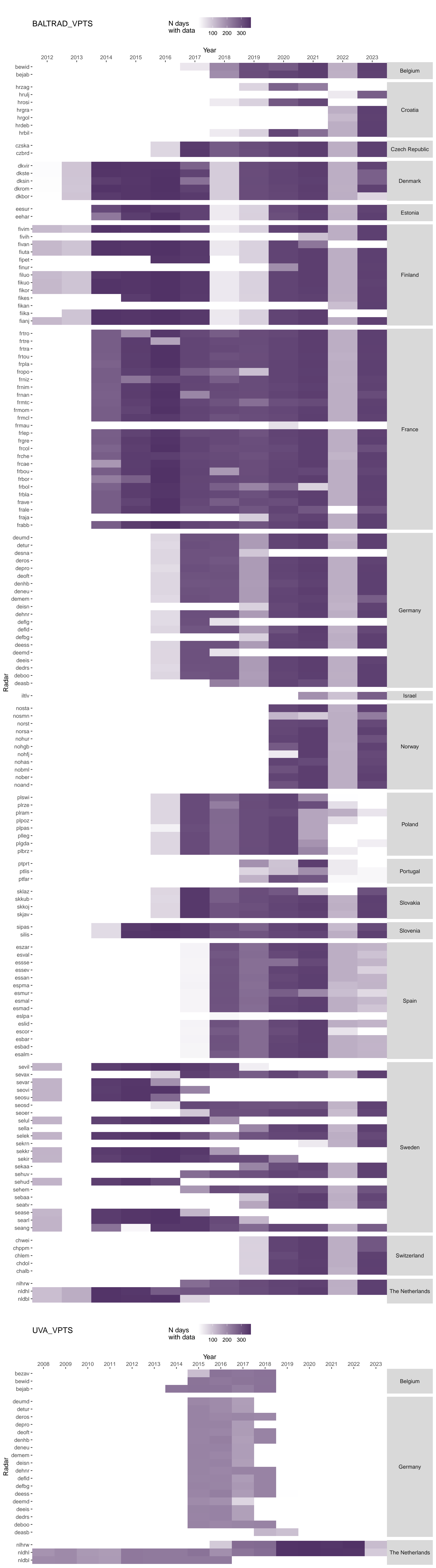

Supplement: Supplementary file 1 — Supplementary Information [file 41597_2025_4641_MOESM1_ESM.pdf]
